# Efficacy of Topical Epinephrine in Preventing Post-Endoscopic Retrograde Cholangiopancreatography Pancreatitis: A Meta-analysis

**DOI:** 10.5152/tjg.2024.23386

**Published:** 2024-09-01

**Authors:** Zhiliang Chen, Hong Fu

**Affiliations:** 1 Department of Hepatobiliary Surgery, Shaoxing People’s Hospital, Shaoxing, China; 2 Shaoxing Key Laboratory of Minimally Invasive Abdominal Surgery and Precise Treatment of Tumor, Shaoxing, China

**Keywords:** Endoscopic retrograde cholangiopancreatography, epinephrine, pancreatitis, meta-analysis

## Abstract

**Background/Aims:**

Post-endoscopic retrograde cholangiopancreatography (ERCP) pancreatitis (PEP) is the most common complication of ERCP. As the clinical effectiveness of topical epinephrine in preventing PEP is elusive, this work attempts to assess its impact on PEP prevention.

**Materials and Methods:**

The databases Embase, Web of Science, PubMed, and Cochrane Library were searched for randomized controlled trials (RCTs) and retrospective cohort studies (RCSs) on topical epinephrine in PEP prevention (data cutoff, November 2022).

**Results:**

This study included a total of 10 research articles, involving 5683 patients, comprising 7 RCTs and 3 RCSs. The results of the meta-analysis indicated that epinephrine had no significant effect on preventing PEP or improving its severity. The meta-analysis results of RCTs subgroup revealed no significant difference in the incidence of PEP between patients receiving epinephrine treatment [alone/in combination with nonsteroidal anti-inflammatory drugs (NSAIDs)] vs. without epinephrine treatment (control group) (*P* = .23). However, patients treated with epinephrine alone experience a lower incidence of PEP compared to the control group (risk ratio [RR] = 0.28, 95% CI = 0.14-0.56, *P* = .0004). The treatment with epinephrine + NSAIDs vs. NSAIDs showed no significant difference (*P* = .95). The meta-analysis results of RCSs subgroup demonstrated a significant reduction in the incidence of PEP with the epinephrine + NSAIDs vs. NSAIDs (*P *< .05). Regarding the severity of PEP [mild, and moderate to severe (M-S)] in the RCT subgroup, the incidence of PEP was not reduced with epinephrine treatment (alone/in combination with NSAIDs) vs. control group. In the RCS subgroup, receiving epinephrine (alone/in combination with NSAIDs) reduced the incidence of mild PEP, while it had no effect on the incidence of M-S PEP.

**Conclusion:**

Epinephrine was not significantly effective in preventing PEP and improving its severity. The combined use of NSAIDs and epinephrine as a possible preventive measure requires further investigation into its efficacy.

**Main Points**

- The incidence of post-endoscopic retrograde cholangiopancreatography (ERCP) pancreatitis (PEP) was lower in patients who received epinephrine treatment than those who did not.
- Patients using epinephrine alone had a lower PEP incidence than those who did not.
- No significant variations in PEP incidence between patients using nonsteroidal anti-inflammatory drugs (NSAIDs) combined with epinephrine and those using NSAIDs alone.
- This study showed that epinephrine had no appreciable impact on preventing PEP or improving PEP severity.

## Introduction

Endoscopic retrograde cholangiopancreatography (ERCP) is frequently used for the detection and treatment of biliary and pancreatic disorders. Post-ERCP pancreatitis (PEP) is the most commonly reported ERCP complication, with an incidence of 2%-10%, exceeding 15% in high-risk patients, and an overall mortality rate of approximately 0.5%-0.9%.^1-3^ PEP has a high incidence and mortality rate due to factors related to both patients and procedures. Patient-related risk factors encompass youthfulness, dysfunction of the Oddi sphincter, and a history of prior PEP. Incubation difficulties or failures, sampling of pancreatic duct tissue, sphincterotomy of the Oddi sphincter, and papillectomy are all surgical risk factors.^4^ Several studies indicate that prophylactic pancreatic duct stenting can reduce the occurrence of PEP, particularly in terms of mitigating the risk of severe PEP. Despite the proven efficacy of pancreatic duct stenting, it requires an experienced endoscopist to minimize stent placement failure.^5,6^ Moreover, pancreatic duct stenting may give rise to significant adverse events, such as intraductal misplacement, inward migration, and pancreatic duct injury, making it an ineffective strategy for treating PEP.^7^

More than 35 drugs, including nonsteroidal anti-inflammatory drugs (NSAIDs), have been assessed for PEP prevention over the years.^8^ NSAIDs, in particular, have been shown in randomized controlled trials (RCTs) to be pivotal in the prevention of PEP.^9^ In a network meta-analysis, Akbar et al^4^ unveiled that rectal NSAIDs were more effective than pancreatic duct stents in controlling post-ERCP pancreatitis. According to a network meta-analysis, rectal NSID-based combination regimens were superior to single regimens in preventing PEP.^10^ Despite using NSAIDs prior to ERCP, PEP occurs in 4%-9% of patients.^9,11^

Apart from NSAIDs, some protease inhibitors (gabexate mesilate, octreotide, ulinastatin, and nafamostat), nitroglycerin, and allopurinol are considered potential medications for the effective prevention of PEP.^8^ However, some of these drugs are expensive, complex, or difficult to administer. For example, octreotide and ulinastatin come with higher costs, and the use of nitroglycerin may be restricted by blood pressure and cardiovascular health, indicating that they are not appropriate for routine clinical use.^12-15^ Given these limitations, there is still a need to investigate drugs with minimal side effects, fewer contraindications, simple administration, and a pronounced preventive effect on PEP.

Xu et al^16^ and Torun et al^12^ found that epinephrine, alone or in combination with NSAIDs, can prevent PEP. But the study by Dar et al^17^ revealed that PEP incidence cannot be reduced by the combination of epinephrine with diclofenac. Rectal NSAIDs and topical epinephrine are found to be the most effective drugs for preventing PEP in a network meta-analysis.^18^ But it included only 2 studies on epinephrine.^16,19^ The findings suggest that epinephrine alone, but not in combination with NSAIDs, may reduce PEP risk. Given the current debate over the efficacy of drugs in preventing PEP, we conducted this systematic review to better assess the role of epinephrine in PEP prevention. Additionally, we investigated its impact on patients with varying degrees of PEP severity.

## Materials and Methods

### Search for Literature

The databases Embase, Web of Science, PubMed, and The Cochrane Library were searched to gather RCTs or retrospective cohort studies (RCSs) on assessing the role of epinephrine in PEP prevention (data cutoff, November 2022). Search terms were as follows: (“cholangiopancreatography, endoscopic retrograde” [MeSH Terms] OR (“cholangiopancreatography” AND “endoscopic” AND “retrograde”) OR “endoscopic retrograde cholangiopancreatography” OR (“endoscopic” AND “retrograde” AND “cholangiopancreatography”)) AND (“epinephrine” [MeSH Terms] OR “epinephrine” OR “adrenalin” OR “adrenaline” OR “epinephrin” OR “epinephrines”) AND (“pancreas” [MeSH Terms] OR “pancreas” OR “pancreatic” OR “pancreatitides” OR “pancreatitis” [MeSH Terms] OR “pancreatitis”).

### Literature Screening

#### Inclusion Criteria:

(1) Subjects had to be ERCP patients over the age of 18; (2) the study had to compare epinephrine to a placebo or a combination of epinephrine and other drugs to the use of other drugs alone; (3) the PEP incidence had to be reported.

#### Exclusion Criteria:

(1) Studies that were published repeatedly or were guidelines, reviews, and case studies were excluded; (2) studies with data inconsistent or could not be extracted were excluded; (3) non-English language literature was excluded.

### Data Extraction and Quality Assessment

Data extraction was done independently by 2 investigators, and disagreements were settled by a third investigator. The following information was excluded from the studies: literature authors, publication year, study design type, drug intervention type (grouping situation), sample size, and pancreatitis incidence rate. The Cochrane Risk of Bias Tool was utilized for quality assessment of included RCTs based on 6 domains: blinding of outcome assessment, blinding of investigators and participants, allocation concealment, random sequence generation, complete outcome data, reporting bias, and other apparent bias, with each domain being scored as high, low, or unclear risk of bias. The quality of cohort studies was assayed with the Newcastle–Ottawa Scale (NOS). The NOS assesses 3 quality parameters (selection, comparability, and outcome) that are divided into 8 specific items. Except for comparability, which can be adjusted based on specific topics of interest, with a maximum score of 2, each item on the scale starts with a score of one. As a result, the maximum score for each study is 9 points, and studies with scores less than 5 are considered to have a high risk of bias.^20^

### Outcome Measure

The primary outcome measure was the incidence of PEP. The severity of PEP was a secondary outcome measure.

### Statistical Analysis

Meta-analysis was completed on Review Manager version 5.4. (The Cochrane Collaboration, 2020) using RR as an indicator of efficacy. The chi-square test was to measure heterogeneity among study results, and the heterogeneity was also tested using *I* ^2^ statistics. If *I* ^2^ ≤ 50% and *P *≥ .1, no statistical heterogeneity was reflected, and a fixed-effects model was applied. Otherwise, statistical heterogeneity was suggested, and a random-effects model was applied.

## Results

### Literature Search Results

**Figure 1.**
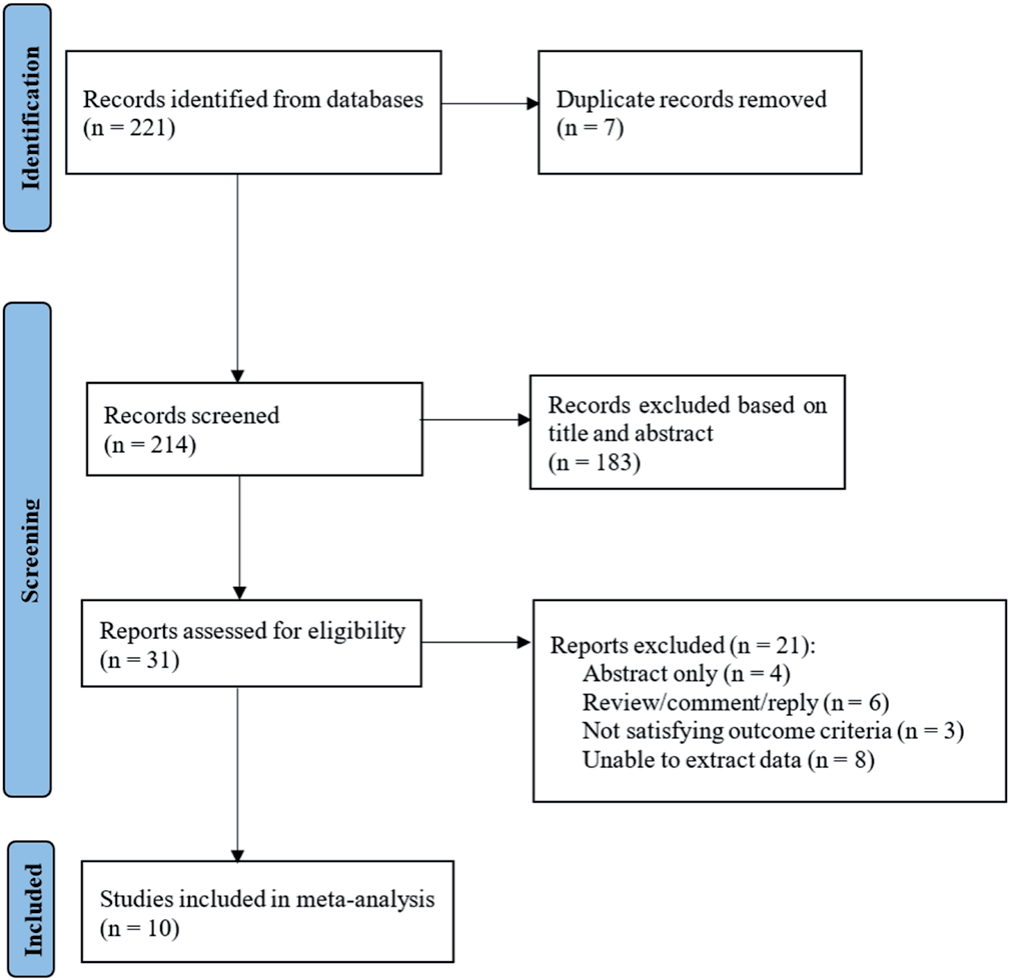
PRISMA flowchart for the included studies.

Relevant studies on the preventive role of epinephrine in post-ERCP pancreatitis were identified through keyword searches in the database using the PRISMA flowchart (Figure 1). A total of 221 studies were initially collected. Following the removal of duplicate studies (7 articles) and the exclusion of irrelevant literature based on title and abstract review, the remaining 31 articles were evaluated for eligibility. Twenty-one of them were excluded due to data unavailability. Ultimately, 10 articles meeting the criteria were included in the study.^12,16,17,19,21-26^

### Characteristics of the Included Studies

Seven of the 10 studies were RCTs^16,17,19,23-25,26^ and 3 were RCSs.^12,21,22^ A total of 5683 patients were involved in the study. Some studies have compared the effectiveness of epinephrine vs. a placebo, while others have evaluated the effects of epinephrine + NSAIDs vs. NSAIDs. The maximum sample size was 941 and the minimum was 126. The characteristics of the literature are detailed in Table 1, which presents specific information on 7 RCTs and 3 RCSs.

**Figure 2.**
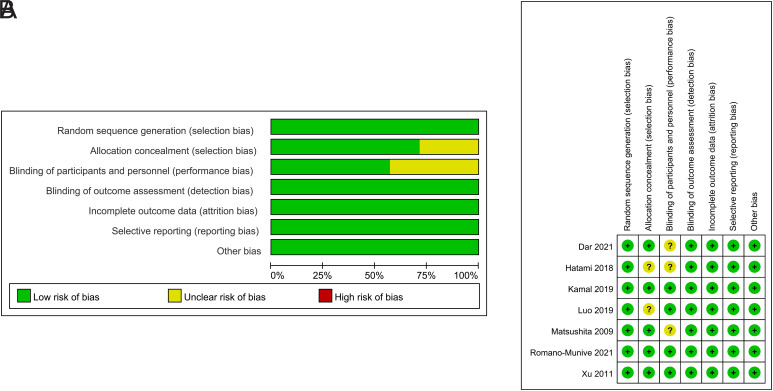
(A) Overall risk of bias; (B) risk of bias for each RCT.

The Cochrane Risk of Bias assessment reported that the risk of bias was low in all included RCTs, and results of the quality assessment were shown in Figure 2A and 2B. Three RCSs had NOS scores greater than 6. Overall, the studies included in this paper are characterized by a low risk of bias and high quality.

### PEP Incidence

**Figure 3.**
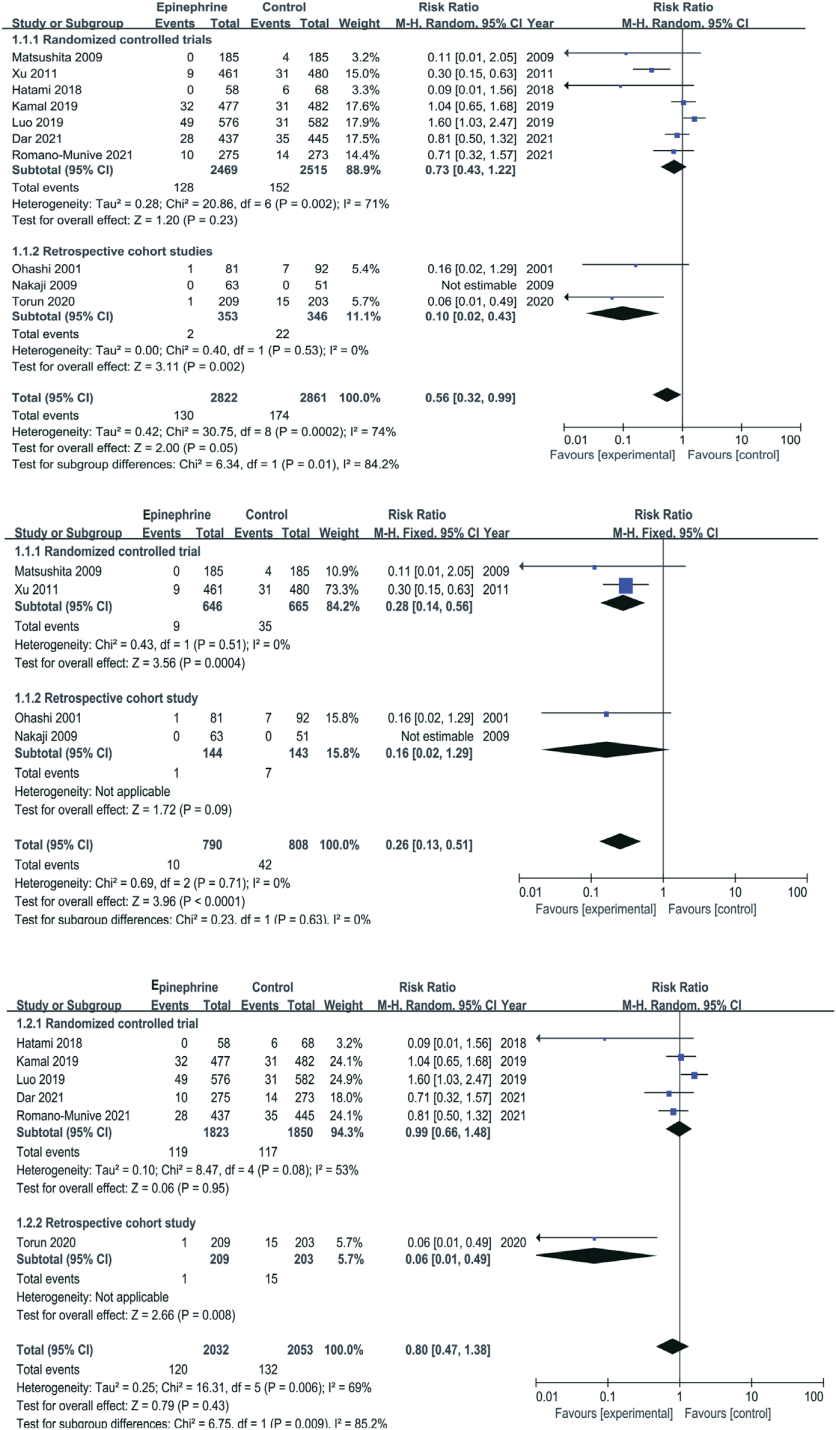
Comparison of the incidence of PEP. (A) Comparison between patients receiving epinephrine treatment and those not receiving epinephrine treatment; (B) Comparison between epinephrine monotherapy and placebo/no-treatment patients; (C) Comparison between the combination of epinephrine and NSAIDs and the use of NSAIDs alone.

The experimental groups, comprising individuals treated with either epinephrine alone or epinephrine + NSAIDs, were compared to the control groups, consisting of patients administered a placebo or NSAIDs. The summarized results are presented in Figure 3A. It was observed that the incidence of PEP was lower in patients with epinephrine treatment (alone/in combination with NSAIDs) vs. without epinephrine treatment (control group). However, the observed difference lacked statistical significance (RR = 0.56, 95% CI = 0.32-0.99, *P* = .05). Subgroup analyses were conducted based on the study type. In the RCTs subgroup analysis, the incidence of PEP showed no difference (RR = 0.73, 95% CI = 0.43-1.22, *P* = .23). However, the RCSs subgroup analysis results were significant (RR = 0.10, 95% CI = 0.02-0.43, *P *= .002).

Four studies^16,19,21,22^ compared the efficacy of using epinephrine alone vs. placebo for preventing PEP (Figure 3B). Patients treated with epinephrine alone had a lower incidence of PEP than those with placebo (RR = 0.26, 95% CI = 0.13-0.51, *P* < .0001). The RCTs subgroup analysis results were consistent with the above, showing significant differences (RR = 0.28, 95% CI = 0.14-0.56, *P *= .0004). However, the RCSs subgroup analysis did not yield significant results (RR = 0.16, 95% CI = 0.02-1.29, *P *= .09).

Six studies^12,17,23-25,26^ compared the effectiveness of epinephrine + NSAIDs vs. NSAIDs for preventing PEP (Figure 3C). The results indicated no significant difference in the incidence of PEP between the 2 groups (RR = 0.80, 95% CI = 0.47-1.38, *P *= .43). The RCTs subgroup analysis results were consistent with the overall findings, showing no difference (RR = 0.99, 95% CI = 0.66-1.48, *P* = .08). However, the RCSs subgroup analysis results were significant (RR = 0.06, 95% CI = 0.01-0.49, *P* = .008).

### Incidence of Mild PEP

**Supplementary Figure 1.**
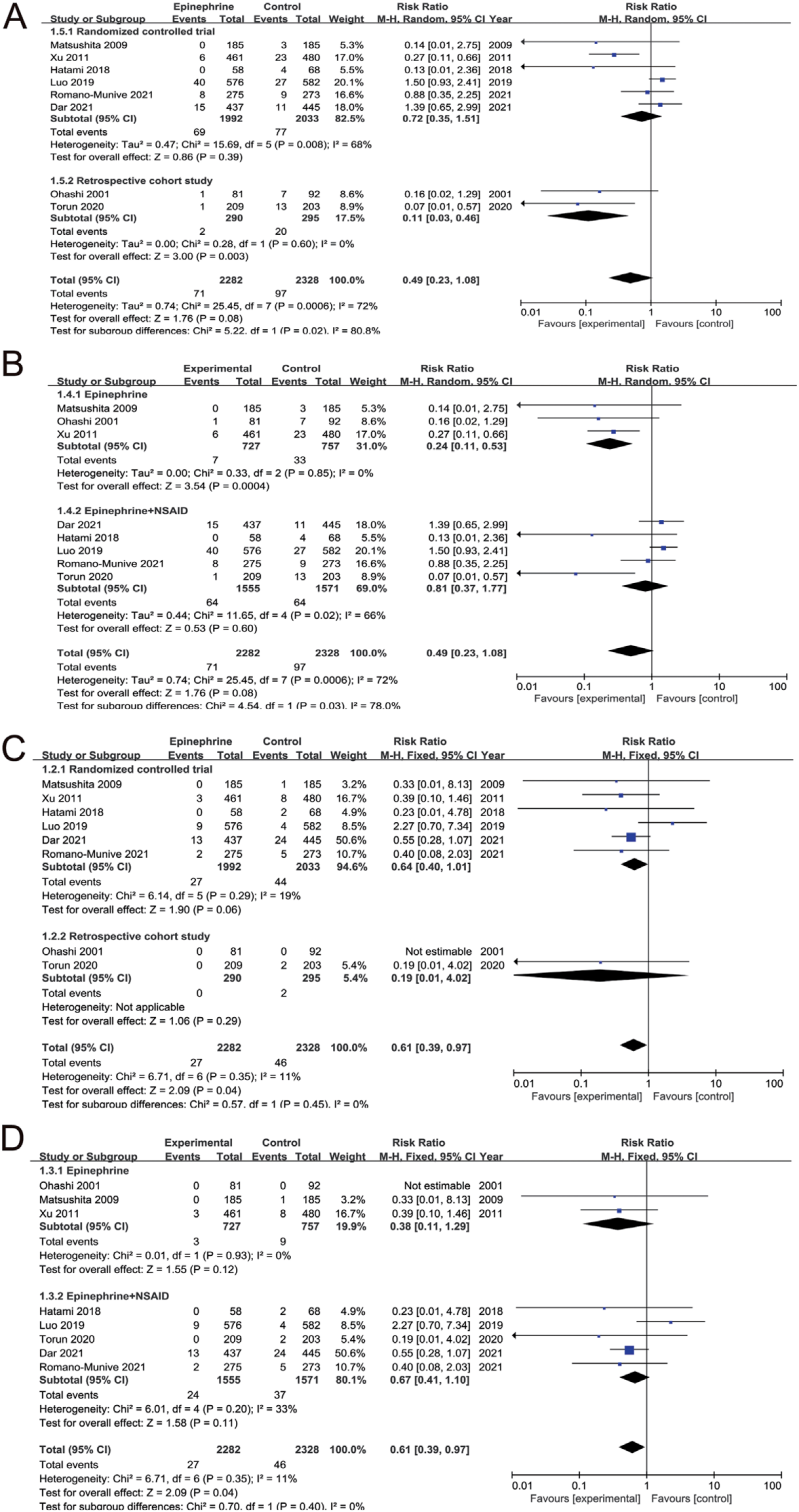
Comparison of the incidence of mild and moderate to severe (M-S) PEP. (A) Comparison of the incidence of mild PEP between patients receiving epinephrine treatment and those not receiving epinephrine treatment; (B) Comparison of the incidence of mild PEP between patients receiving epinephrine monotherapy and placebo/no-treatment patients, as well as the between the combination of epinephrine and NSAIDs and the use of NSAIDs alone; (C) Comparison of the incidence of M-S PEP between patients receiving epinephrine treatment and those not receiving epinephrine treatment; (D) Comparison of the incidence of M-S PEP between patients receiving epinephrine monotherapy and placebo/no-treatment patients, as well as the between the combination of epinephrine and NSAIDs and the use of NSAIDs alone.

Eight studies^12,16,17,19,21,23,24,26^ reported the incidence of mild PEP. The summarized results are presented in Supplementary Figure 1A. It was observed that patients with epinephrine treatment (alone/in combination with NSAIDs) had a lower incidence of mild PEP compared to those without epinephrine treatment (control group), but the observed difference lacked statistical significance (RR = 0.49, 95% CI = 0.23-1.08, *P* = .08). The RCTs subgroup analysis results were consistent with the overall findings, showing no significant difference (RR = 0.72, 95% CI = 0.35-1.51, *P* = .39). However, the RCSs subgroup analysis indicated that epinephrine treatment could reduce the incidence of mild PEP (RR = 0.11, 95% CI = 0.03-0.46, *P* = .003).

Furthermore, 3 studies^16,19,21^ compared the efficacy of using epinephrine alone vs. placebo for preventing mild PEP Supplementary Figure 1B. The results showed that patients with epinephrine alone had a lower incidence of mild PEP (RR = 0.24, 95% CI = 0.11-0.53, *P* = .0004). In addition, 5 studies^12,17,23,24,26^ compared the preventive effects of epinephrine + NSAIDs vs. NSAIDs Supplementary Figure 1B. The 2 groups showed no significant difference (RR = 0.81, 95% CI = 0.37-1.77, *P* = .60).

### Incidence of Moderate-to-Severe PEP

Eight studies^12,16,17,19,21,23,24,26^ presented the incidence of M-S PEP. The summarized results are presented in Supplementary Figure 1C. It was observed that patients with epinephrine treatment (alone/in combination with NSAIDs) had a lower incidence of M-S PEP compared to those without epinephrine treatment (control group) (RR = 0.61, 95% CI = 0.39-0.97, *P* = .04). However, subgroup analyses for RCTs and RCSs both showed no significant difference in the incidence of M-S PEP between the 2 groups (> .05).

Furthermore, 3 studies^16,19,21^ compared the preventive effects of using epinephrine alone vs. placebo for M-S PEP Supplementary Figure 1D. The results revealed no significant difference in the incidence of M-S PEP between the 2 groups (RR = 0.38, 95% CI = 0.11-1.29, *P* = .12). Additionally, 5 studies^12,17,23,24,26^ compared the preventive effects of epinephrine + NSAIDs vs. NSAIDs for M-S PEP Supplementary Figure 1D, and again, the 2 groups showed no significant difference (RR = 0.67, 95% CI = 0.41-1.10, *P* = .11).

## Discussion

We analyzed epinephrine for PEP prevention in 5,683 ERCP patients using data from 10 studies. When compared to patients not treated with epinephrine, our findings suggested that patients treated with epinephrine alone had a lower PEP incidence and could effectively reduce the incidence of mild PEP. However, combination of epinephrine and NSAIDs did not have significant effect in preventing incidence of PEP or improving M-S PEP.

PEP is a well-known ERCP complication that can adversely affect patients’ quality of life and increase their mortality rate. The ASGE and ESGE have issued guidelines to reduce the incidence and severity of PEP, which include prophylactic medications and pancreatic stenting.^27,28^ Adrenaline, NSAIDs, hydration, protease inhibitors, and pancreatic enzyme secretion inhibitors are examples of preventive medications. Choi et al^29^ investigated the impact of perioperative aggressive intravenous fluid resuscitation (IVFR) on the prevention of PEP. The results indicated that IVFR combined with LRS had a preventive effect on both high-risk and moderate-risk cases, reducing the severity of PEP. Park et al^30^ compared the effects of ulinastatin and nafamostat in preventing PEP. The results showed that the incidence of PEP was 1.9% and 3.8%, respectively, indicating that both drugs reduced the incidence of PEP. Furthermore, the placement of a prophylactic pancreatic stent (PSP) can reduce the risk of PEP by alleviating the pancreatic duct hypertension caused by surgery-induced edema and pancreatic duct stricture.^31,32^ PSP can significantly reduce the incidence of mild, moderate, and severe pancreatitis in high-risk patients, according to a meta-analysis of RCTs.^33,34^ The efficacy of PSP in preventing PEP was investigated by comparing it to a control group that received non-stent procedures, and the results showed a lower incidence of PEP with PSP.^35^ Additionally, the length of pancreatic stents affects their effectiveness in preventing PEP. A prospective randomized trial compared the efficacy of 3 cm and 5 cm pancreatic stents in preventing PEP, revealing that the 3 cm stent outperforms the 5 cm stent.^36^

Akshintala et al^18^ conducted a comparative and ranking analysis of drugs for the direct and indirect prevention of PEP using Bayesian network meta-analysis. The results revealed that topical adrenaline is the most effective drug for preventing PEP (OR: 0.25, 95% CI 0.06-0.66) and has been recommended by the American Society for Gastrointestinal Endoscopy (ASGE) guidelines. The proposed mechanism of action involves vasoconstriction mediated by adrenaline, leading to the constriction of small arterial vessels in the papillary mucosa. This, in turn, results in the direct relaxation of the Oddi sphincter and a reduction in capillary permeability, thereby decreasing papillary edema and subsequent pancreatic duct outflow obstruction to prevent PEP.^16,19,25,37^ Matsushita et al^19^ conducted the first RCT on epinephrine for PEP prevention. They observed a reduced PEP incidence in the epinephrine group (0/185) compared to control group (4/185) (*P *= .12). But the sample size was called into question. Another RCT by Xu et al^16^ observed a reduction in PEP incidence in the epinephrine group (9/461) as compared with the control group (31/480) (*P *= .008). Our meta-analysis revealed that patients who used epinephrine alone had a lower incidence of PEP than those who did not. However, due to high heterogeneity among included studies, we performed a subgroup analysis. Results showed that in RCTs, topical use of epinephrine significantly decreased PEP incidence, while in RCSs, epinephrine use had no significant preventive impact on PEP. Variations in meta-analysis results may be attributed to differences in study design types. For instance, there is a potential bias in subject selection in the RCSs subgroup. Despite the lower heterogeneity, the number of patients included in this study is relatively small. In addition, the results of RCTs subgroup analysis were not significant, which could be related to the high heterogeneity caused by the large differences in drug groups and sample size. To further investigate the preventive effect of epinephrine on PEP severity, we conducted a categorical analysis of the included studies. Firstly, local epinephrine treatment had no significant impact on overall incidence of mild PEP. In mild PEP, the pooled results of the meta-analysis show that epinephrine treatment has no significant impact on the overall incidence rate of PEP (*P *= .08). However, in the subgroup analysis with a study design of RCSs, a significant decrease in the incidence rate of mild PEP is observed (*P *= .003). In M-S PEP, the use of epinephrine significantly reduces the incidence rate of PEP (*P* = .04).

NSAIDs are recommended for the prevention of PEP, primarily by rectal administration of diclofenac or indomethacin prior to or after ERCP, and RCTs have confirmed this conclusion.^9,38^ However, recent publications on the effectiveness of the combination of NSAIDs and topical epinephrine spray at the duodenal papilla for preventing PEP have yielded conflicting results.^23,25,39-42^ In a recent double-blind clinical trial involving 164 patients, it has been found that combination therapy is more effective than the sole use of indomethacin, with incidence rates of 2.4% and 4.9%, respectively.^43^ Conversely, in a multicenter double-blind randomized controlled study, Kamal et al^25^ have discovered that the combined use of epinephrine and NSAIDs (indomethacin) does not reduce the incidence of PEP compared to the use of NSAIDs alone (6.4% vs. 6.7%). In a large-scale (1158 cases) double-blind trial conducted in China (ClincialTrials.gov number: NCT03057769), the combination therapy of epinephrine and NSAIDs (indomethacin) has been found to increase the risk of PEP.^17^ Therefore, our study included literature from both RCTs and RCSs to analyze the preventive effects of epinephrine and NSAIDs, either alone or in combination, on PEP. Our results indicated that, compared to the sole use of NSAIDs, the combination of epinephrine and NSAIDs was ineffective in preventing the incidence of PEP. Three possible reasons can explain our findings. Firstly, the mechanism by which epinephrine prevents PEP is by inducing vasoconstriction at the level of the duodenal papilla by binding to α-1 receptors,^25,44^ thereby reducing tissue edema around the pancreatic duct (PD) opening to prevent PEP.^18,22^ However, the spray of epinephrine may lead to reduced blood supply, diminishing the local concentration of NSAIDs (indomethacin/diclofenac) in pancreatic tissue, counteracting the beneficial effects of NSAIDs.^18^ Secondly, there may be an interaction between topical epinephrine and NSAIDs. NSAIDs prevent PEP by inhibiting phospholipase A2 activity and reducing inflammation.^45-47^ However, some studies suggest that epinephrine activates phospholipase A2 by stimulating Na+/H+ antiporters.^47-49^ Thirdly, NSAIDs have a wide impact on various tissues, which could interfere with the action of topical epinephrine, rendering them ineffective.^50^ Further research is needed to comprehensively evaluate the biological distribution of NSAIDs with or without epinephrine in human or animal subjects, shedding light on their mechanisms of action.

The study has some limitations. First, 3 studies are RCSs, and the results of their subgroup analysis are more significant than those of the RCTs subgroup analysis, which may introduce bias. Second, because of the small number of included studies, some results for RCTs and RCSs subgroups could not be analyzed, potentially leading to bias in meta-analysis results. Third, the clinical characteristics of patients included in the study, particularly the combination of drugs, dose and concentration of drugs used, the definition of PEP, and the definition of severity, were different, resulting in study heterogeneity. Fourth, some studies lacked data on high-risk PEP patients, and more studies are needed for further analysis of subgroup analysis of severity. Fifth, patients with preventive placement of pancreatic stents were excluded from the study, which could be one of the factors influencing heterogeneity.

In summary, this study found that epinephrine had no significant impact on preventing PEP or improving the severity of PEP. Prospective research with large sample sizes is required to confirm the effects of epinephrine in different contexts of use.

## Figures and Tables

**Table 1.** Characteristics of Studies Included in this Meta-Analysis

Author	Year	Study Design	Intervention		Sample Size (n)	
			Study	Control
Study	Control
Ohashi	2001	RCS	Epinephrine	No treatment	81	92
Matsushita	2009	RCT	Epinephrine	Saline spray	185	185
Nakaji	2009	RCS	Epinephrine	No treatment	63	51
Xu	2011	RCT	Epinephrine	Saline spray	461	480
Hatami	2018	RCT	Indomethacin + epinephrine	Indomethacin	58	68
Luo	2019	RCT	Indomethacin + epinephrine	Indomethacin + saline spray	576	582
Kamal	2019	RCT	Indomethacin + epinephrine	Indomethacin + saline spray	477	482
Torun	2020	RCS	Indomethacin + epinephrine	Indomethacin	209	203
Dar	2021	RCT	Diclofenac + epinephrine	Diclofenac + saline spray	437	445
Romano-Munive	2021	RCT	Indomethacin + epinephrine	Indomethacin + saline spray	275	273
